# Introduction of Rare-Earth Oxide Nanoparticles in CNT-Based Nanocomposites for Improved Detection of Underlying CNT Network

**DOI:** 10.3390/nano11092168

**Published:** 2021-08-25

**Authors:** Joel Hubbard, Tugba Isik, Troy Y. Ansell, Volkan Ortalan, Claudia Luhrs

**Affiliations:** 1 Mechanical and Aerospace Engineering Department, Naval Postgraduate School, Monterey, CA 93943, USA; troy.ansell@nps.edu (T.Y.A.); ccluhrs@nps.edu (C.L.); 2 Materials Science and Engineering Department, University of Connecticut, Storrs, CT 06269, USA; tugba.isik@uconn.edu (T.I.); vortalan@uconn.edu (V.O.); 3 School of Mechanical Engineering, Purdue University, West Lafayette, IN 47907, USA

**Keywords:** carbon nanotubes, Eu:Y_2_O_3_, epoxy-CNT composites, micro-CT

## Abstract

Epoxy resins for adhesive and structural applications are widely employed by various industries. The introduction of high aspect ratio nanometric conductive fillers, i.e., carbon nanotubes, are well studied and are known to improve the electrical properties of the bulk material by orders of magnitude. This improved electrical conductivity has made carbon nanotube-based nanocomposites an attractive material for applications where their weight savings are at a premium. However, the analytical methods for validating carbon nanotube (CNT) nanofiller dispersion and for assuring that the properties they induce extend to the entire volume are destructive and inhibited by poor resolution between matrix and tube bundles. Herein, rare-earth oxide nanoparticles are synthesized on CNT walls for the purpose of increasing the contrast between their network and the surrounding matrix when studied by imaging techniques, alleviating these issues. The adherence of the synthesized nanoparticles to the CNT walls is documented via transmission electron microscopy. The crystalline phases generated during the various fabrication steps are determined using X-ray diffraction. Deep ultraviolet-induced fluorescence of the Eu:Y_2_O_3_-CNT nanostructures is verified. The impacts to nanocomposite electrical properties resulting from dopant introduction are characterized. The scanning electron microscopy imaging of CNT pulp and nanocomposites fabricated from untreated CNTs and Eu:Y_2_O_3_-CNTs are compared, resulting in improved contrast and detection of CNT bundles. The micro-CT scans of composites with similar results are presented for discussion.

## 1. Introduction

Nanoscale composites utilizing carbon nanotube (CNT) filler allow for significant reductions in resistivity of an otherwise highly insulating matrix material while requiring extremely low CNT loadings [1]. These improved conductivities result in an attractive material with extensive applications in diverse industries, but particularly for electrostatic discharge (ESD) and electromagnetic interference (EMI) prevention [2,3,4,5,6,7,8,9,10,11]. These enhancements are heavily dependent on nanofiller dispersion in the matrix material. Dispersion control becomes essential to ensuring uniform properties throughout the composite and eliminating hotspots that could affect reliability or performance.

Validation of uniform dispersion is difficult due to minimal contrast differences between CNTs and the surrounding matrix, making conventional imaging techniques difficult. Existing high-resolution methods include thermography in the infrared (IR) range [12] and voltage-contrast imaging with scanning electron microscopy (SEM) [13,14,15,16,17,18]. Both have been used to understand dispersion and nanostructures but are limited by the processing time and low fields-of-view. Thermography techniques are depth limited as the subsurface information is extrapolated.

Nondestructive evaluation (NDE) techniques that make the differences in the dispersion of the nanofiller or the porosity of the surrounding matrix readily apparent would be useful as the industrial use of these composites increases. This work aimed to improve the contrast of the CNT network through the addition of rare-earth oxides, specifically europium doped yttria, to the CNT surface. Europium doped yttria has been found to emit red light under stimulation by deep ultraviolet (DUV) wavelengths [19,20]. Since its discovery, Eu:Y_2_O_3_ has become a common red phosphor material due its narrow emission peak, easy stimulation under DUV radiation, and high quantum efficiency [21,22,23,24]. Efforts to exploit the demonstrated luminescence for increased visibility in bioimaging have been explored through the use of Eu:Y_2_O_3_ nanorods [25] and nanoparticles incorporated into CNT cavities [26]. Coincidently, rare-earth elements are heavier than those present in a conventional CNT composite, e.g., iron, carbon, and polymer chains. These heavier elements facilitate the compositional contrast through backscatter electron production in SEM imaging. By colocating Eu:Y_2_O_3_ nanoparticles and CNTs (hereafter Eu:Y_2_O_3_-CNT), the detection of red-emissions or the detection of heavy elements, yttrium and europium, can reveal the location of an underlying CNT network and make bulk dispersion determinations of the CNT filler easier to visualize and at greater depths.

## 2. Materials and Methods

### 2.1. Materials

The CNT pulp that served as substrate for the Eu:Y_2_O_3_ nanoparticle synthesis, and for the epoxy composite fabrication, was a multiwall carbon nanotube (MWCNT) product provided by Nanocomp Technologies Inc. (Huntsman Corporation, Merrimack, NH, USA). This nanofiller is produced as CNT sheets via a chemical vapor deposition (CVD) process facilitated by an iron (Fe) catalyst and subsequently reduced to a pulp by employing a Hollander Beater and industrial burr [27]. The pulp consists of MWCNT bundles approximately 0.05 mm in diameter and 1 mm in length with individual CNT diameter in the order of 30 nm. The pulp used throughout this research comes from a single production run (Lot C) and is contrasted later with CNTs from past batches (Lot A,B).

Yttrium (III) and europium (III) nitrate hexahydrate obtained from Sigma Aldrich (St. Louis, MO, USA) with a purity of 99.8% and 99.99% respectively, were used as precursors and dissolved in a >99.5% pure ethanol also obtained from Sigma Aldrich (St. Louis, MO, USA).

### 2.2. Carbon Nanotube Preparation and Eu:Y_2_O_3_ Nanoparticle Synthesis

Sample generation consisted of a three-step process where CNTs were (1) activated, (2) Eu:Y_2_O_3_ synthesized along tube walls, and (3) the resulting pulp dispersed into an epoxy matrix to form the nanocomposite.

**Figure 1:**
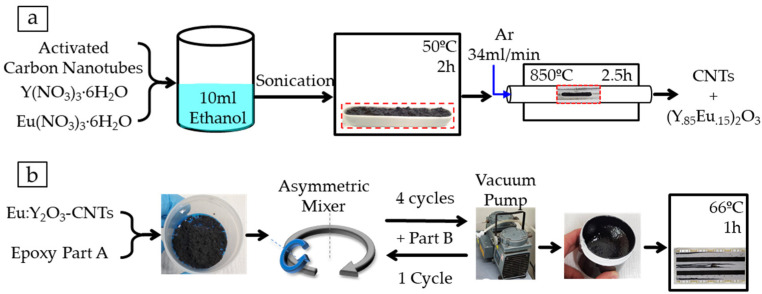
Depiction of the processes for: (**a**) introducing Eu:Y_2_O_3_ nanoparticles to CNT walls; (**b**) fabrication of composite electrical boards and samples from epoxy and CNT pulp.

The CNT pulp was first activated to facilitate the nucleation of rare-earth oxides at activation sites. CNTs were placed in an alumina boat and thermally activated by heating them to 500 °C in a tube furnace (Thermo Scientific Lindberg/Blue M model TF55035A-1, Waltham, MA, USA) for either 1, 2, or 2.5 h in an air atmosphere. The europium and yttrium nitrates were mixed into the activated CNT bundles via wet impregnation aided by the sonication technique illustrated in Figure 1a. Yttrium (III) and europium (III) nitrate hexahydrate and the activated CNT pulp were weighed to achieve europium substitution within the Y_2_O_3_ with a targeted stoichiometry of (Y_0.85_Eu_0.15_)_2_O_3_ and a 5%wt of Eu:Y_2_O_3_ in the CNT pulp. The nitrates were dissolved in 10 mL of ethanol and the resulting solution poured over the CNT pulp and agitated in an ultrasonic bath for several seconds. The saturated CNT bundles were dried at 50 °C for 2 h in a convection oven (Binder GmbH, Tuttlingen, Germany). Once dried, the remaining pulp and the alumina boat were placed in the tube furnace and purged with argon for 30 min. The mixture was calcinated in an ultrahigh purity Ar atmosphere for 2.5 h at 850 °C. The pulp was allowed to cool to room temperature over ~3 h under Ar.

### 2.3. Nanocomposite Fabrication

The nanocomposites used throughout this study were synthesized using the CNT pulp described in Section 2.1 and a two-part aerospace-grade epoxy matrix, Loctite Hysol EA 9396 (Henkel Corporation, Dusseldorf, Germany). The two-part epoxy was employed at a ratio of 100:30 by weight (part A:part B). All samples were cured at 66 °C for 1 h. The base epoxy mixture had a reported electrical resistivity of 2.14 × 10^15^ Ohm-cm, tensile strength of 35.2 MPa, and lap shear strength of 27.6 MPa at 25 °C [28]. 

The CNT dispersion and electrical board fabrication is illustrated in Figure 1b. The epoxy and CNTs were weighed to generate the desired loading (0.75%wt) and then dispersed by mixing in a FlackTek asymmetric speed mixer (Landrum, SC, USA). The mixer was operated for 3 min at lower speeds followed by three 1 min higher-speed cycles with Part B added before the final cycle. A vacuum pump was used to evacuate the air from the hermetic vessel where the samples were contained (low vacuum range) throughout the mixing process. The mixture was then applied to prefabricated electrical boards and, separately, poured into molds for curing. A convection oven (Binder GmbH, Tuttlingen, Germany) was used for the 66 °C curing process.

### 2.4. Thermo-Gravimetric Analysis

Untreated CNT pulp was analyzed in a simultaneous thermal analyzer (STA) 449 Jupiter F1 (Netzsch GmbH & Co. Holding KG, Selb, Germany) to determine initial iron catalyst content. Data was collected during a temperature-programmed oxidation, heating the CNTs under a 20% O_2_ 80% N_2_ environment analogous to sea-level atmosphere composition, at a rate of 15 °C/min from room temperature to 850 °C.

### 2.5. Material Characterization and Imaging

A benchtop powder X-ray diffraction (XRD) Rigaku Miniflex 600 (Woodlands, TX, USA) with a Cu-Source was used to determine crystalline phases throughout the process of activation and Eu:Y_2_O_3_ inclusion.

An optical metallurgical microscope Nikon Epiphot 200 (Melville, NY, USA) was employed for low magnification observation of the CNT dispersions within the epoxy matrix.

A Zeiss Neon 40 (Zeiss, Oberkochen, Germany) Dual Beam SEM operating from 2 to 20 KV was utilized to investigate microstructural features of untreated CNT and Eu:Y_2_O_3_-CNT bundles and the resulting CNT epoxy composites. Secondary electron (SE) and backscattered electron (BSE) images were collected.

High-resolution transmission electron microscopy (HRTEM) images were acquired using a FEI Tecnai Osiris TEM (Hillsboro, OR, USA). TEM bright-field images were captured with an accelerating voltage of 200 kV, a second condenser aperture of 50 µm, and an objective aperture of 40 µm. Scanning TEM (STEM) was used in conjunction with a high angle annular dark-field (HAADF) detector for energy dispersive X-ray (EDX) spectroscopy studies, the TEM’s coupled Super-X system.

Micro-computed tomography (micro-CT) was utilized on two composite samples to determine if improvements in network detection were possible with Eu:Y_2_O_3_-CNT samples. The analysis was performed with a Zeiss Xradia 520 Versa (Zeiss, Oberkochen, Germany). Scan parameters were 100 kV source voltage, 9 W power, and 10× optical magnification. The source and detector distances were adjusted to achieve a voxel size of approximately 0.7 µm^3^ and 1601 projections through 360 degrees were collected with an exposure time of 1 s. Three-dimensional (3D) tomograms were rendered using tomviz freeware [29].

### 2.6. Deep Ultraviolet Fluorescence

Photoluminescence measurements of Eu:Y_2_O_3_-CNT composite samples were conducted using a LabRAM HR Raman Microscope (Horiba Scientific, Kyoto, Japan). A deep ultraviolet (DUV) source with an emission wavelength of 229 nm was used with a 600 gr/nm grating. Source power was limited to 100 µW due to sample degradation resulting in broadening of spectra peaks at higher intensities.

### 2.7. Conductivity Characterization

**Figure 2:**
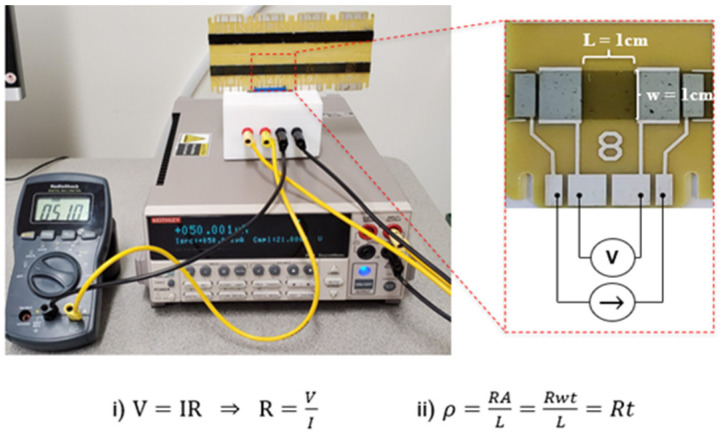
Experimental setup for measure electrical resistivity and governing equations.

All electrical measurements were done by analyzing the prefabricated eight-point circuit boards with a 2400 Keithley source meter (Tektronix, Inc., Beaverton, OR, USA) operating as a current source and can be seen in Figure 2. A separate digital multimeter (DMM) was used to measure the voltage drop across the 1 cm wide epoxy section. One spot location was sectioned, and the thickness was measured via a Nikon Epiphot 200 reflective optical microscope (Nikon, Tokyo, Japan) to determine an average film thickness. The electrical resistivity was then derived using Equation (ii) in Figure 2.

## 3. Results and Discussion

### 3.1. Characterization of CNT Pulp and Synthesized Eu:Y_3_O_3_ Nanoparticles

**Figure 3:**
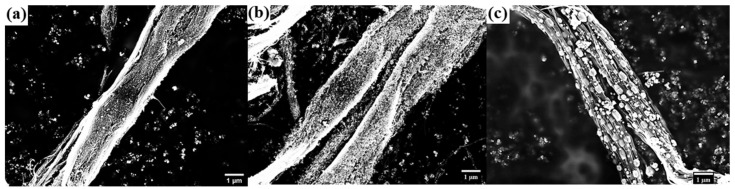
SEM imaging of CNT bundles at 10k× magnification: (**a**) untreated CNT bundles; (**b**) activated bundles; (**c**) Eu:Y_2_O_3_-CNT bundles.

Scanning electron microscopy observations of untreated, activated, and Eu:Y_2_O_3_-CNT pulp were conducted and are shown in Figure 3. The untreated pulp shown in Figure 3a consisted of tight bundles of CNTs, in contrast with Figure 3b, where the loosely packed bundles of CNTs activated for 2.5 h can be seen. Figure 3c shows a section of the Eu:Y_2_O_3_-CNT pulp that exemplified a feature observed in all sample locations; increased laxity of the bundle structure. This progressive breakdown of the bundled CNTs results from the mechanical agitation associated with handling and sonication during the activation and Eu:Y_2_O_3_ nanoparticle synthesis.

**Figure 4:**
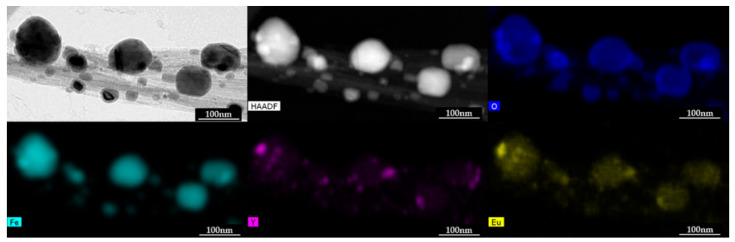
HRTEM, HAADF, imaging and EDX mapping of CNT bundles showing elemental distribution.

**Figure 5:**
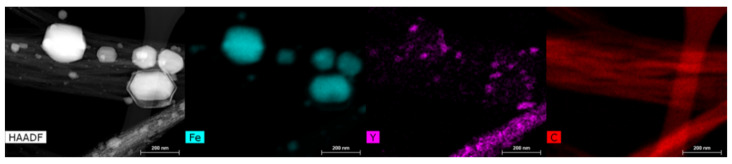
HAADF imaging and EDX mapping of CNT bundles showing the Yttria distribution on CNT wall.

HRTEM analysis was conducted to determine the location of the synthesized Eu:Y_2_O_3._ In all cases, the rare-earth oxides were found adhered to CNT bundles or to surrounding Fe particulates, never as individual particulates. CNT bundles were found to have Eu:Y_2_O_3_ nanoparticles of approximate diameters from 3 to 20 nm adhered to the tube or Fe surface despite the use of sonication to disperse the sample for observation. HRTEM analysis with the corresponding EDX elemental maps for Fe, Y, Eu, and oxygen are presented in Figure 4. Figure 5 shows a study performed in a different section of the sample, and is included to point out that the Eu:Y_2_O_3_ distribution in the samples occurred throughout the CNT network, to include both the Fe catalyst and bare CNT surfaces.

Iron particles, internal and external to the CNTs, with diameters between 10 and 120 nm, could be seen in all the sections analyzed and are a remnant of the CNT growth process that utilized iron as a catalyst. Larger iron particles > 30 nm were found on the tube surface and were surrounded by an oxide shell of approximately 5–8 nm in thickness. Synthesized europium-yttrium oxide particulates were distributed throughout the CNT and Fe surfaces and as agglomerated nodules reaching 20 nm in diameter on larger iron particles. This colocation of Eu:Y_2_O_3_ nanoparticles along the tube walls ensures that their detection indicates the presence of an underlying CNT bundle. It is worth noting that some of the Fe particulates appeared to sinter during the step meant to decompose the nitrates, conducted at 850 °C and described in Section 2.3.

**Figure 6:**
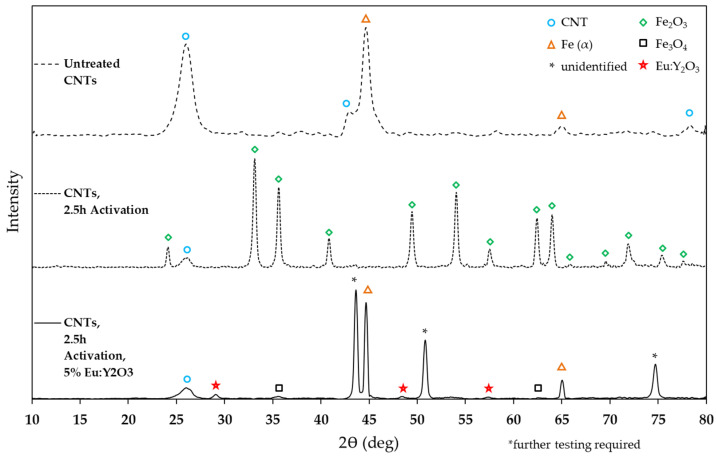
XRD analysis of untreated, activated, and Eu:Y_2_O_3_-CNT pulp with corresponding phases identified.

X-ray diffraction was conducted with untreated CNTs, CNT pulp activated for 2.5 h, and the final product of 5wt% Eu:Y_2_O_3_-CNT pulp. Figure 6 presents the three samples with significant phases identified.

Untreated CNT pulp contained the ferritic iron used as a catalyst during the growth process and was left in place. Subsequent TGA analysis on the same pulp determined the iron content to be 25.6% by weight. Activation of the untreated pulp resulted in complete oxidation of the iron to form iron (III) oxide, Fe_2_O_3_. The Eu:Y_2_O_3_-CNT pulp sample exhibited iron and a new iron oxide phase, Fe_3_O_4._ The Eu:Y_2_O_3_ synthesis process introduced europium and yttrium nitrate hydrate and required calcination. These precursors underwent thermal decomposition of the hydrates and off-gassing of oxynitrates in various stages before eventually decomposing into the target oxides between 600 and 700 °C [30,31]. This same process results in the reduction of Fe_2_O_3_ to Fe_3_O_4_ and iron. The peaks identified as Eu:Y_2_O_3_ are consistent with JCPDS #058-0800 where similar Eu doping of the Y_2_O_3_ lattice was conducted as (Y_0.8_Eu_0.2_)_2_O_3_.

A phase was also identified in the completed Eu:Y_2_O_3_, marked in Figure 6 by an asterisk symbol. Two potential candidates were identified, iron nitrides with stoichiometric close to FeN_x_ (with 0.03 < x < 0.09) or a carbon allotrope referred to as n-diamond (new-diamond). Previous studies have cataloged peaks with these iron nitrides [32]. However, data has suggested that pure single phases where x < 0.2 have not been observed and are energetically unfavorable [33]. N-diamond was discovered by Hirai and Kondo [34] at high temperatures and pressures and has since been synthesized by numerous groups in varying conditions [35]. The most relevant by Wen et al. using iron-catalyzed CNTs at atmospheric pressures and temperatures as low as 800 °C [36] at the sacrifice of the degradation of the CNT structure—something not seen here. Further analyses are required to unequivocally differentiate the phase that gives origin to these peaks.

In sum, the XRD analysis confirmed the presence of Eu:Y_2_O_3_ and TEM identified it as colocated with the CNT network.

**Figure 7:**
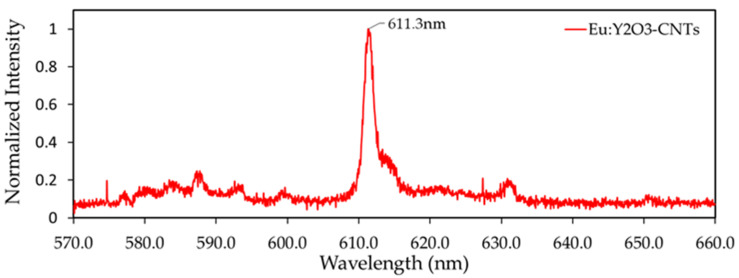
Photoluminescent spectra resulting from 229 nm excitation of 5%wt Eu:Y_2_O_3_ CNTs that had been activated for 2.5 h.

The Eu^3+^ doping of yttria produces known photoluminescence in the visible spectrum. Figure 7 shows the emission spectra of Eu:Y_2_O_3_-CNTs under an excitation wavelength of 229 nm. The peak at 611 nm is the characteristic spectra indicative of Eu^3+^ transition from ^5^D_0_ → ^7^F_2_ that results in the desired red luminescence [19,20,23] and confirms the successful introduction of Eu^3+^ ions into the host Y_2_O_3_ lattice.

### 3.2. Characterization of the CNT and Eu:Y_2_O_3_-CNT Composites and Their Electrical Performance

**Figure 8:**
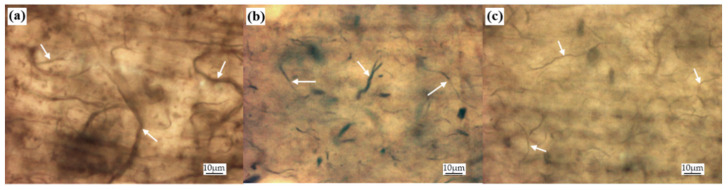
Magnification microscope 50× images revealing CNT bundles, indicated by white arrows, in nanocomposites created from: (**a**) untreated CNT pulp; (**b**) 2.5 h activated CNT pulp; (**c**) Eu:Y_2_O_3_-CNTs.

Optical microscopy observations of epoxy nanocomposite films can be seen in Figure 7. The three-dimensional network of CNT bundles is comprised of bundles of increasingly smaller diameter and length at each step of the Eu:Y_2_O_3_-CNT production process. Under SEM imaging, the CNT pulp in Figure 3 exhibited laxation of the CNT bundle due to mechanical agitation. Dispersing the CNT pulp in the epoxy was done via a dual asymmetric mixer. It is suspected that the shear forces resulting from mixing in epoxy further separate the CNT bundles resulting in the smaller mean bundle size seen in Figure 8c.

Observationally, significant decreases in viscosities were seen between the uncured samples and support the previous discussion. The uncured epoxy with untreated CNT filler must be manually deposited into a mold and has a distinct texture, while the uncured Eu-Y_2_O_3_-CNTs epoxy is smooth and can be readily poured.

**Figure 9:**
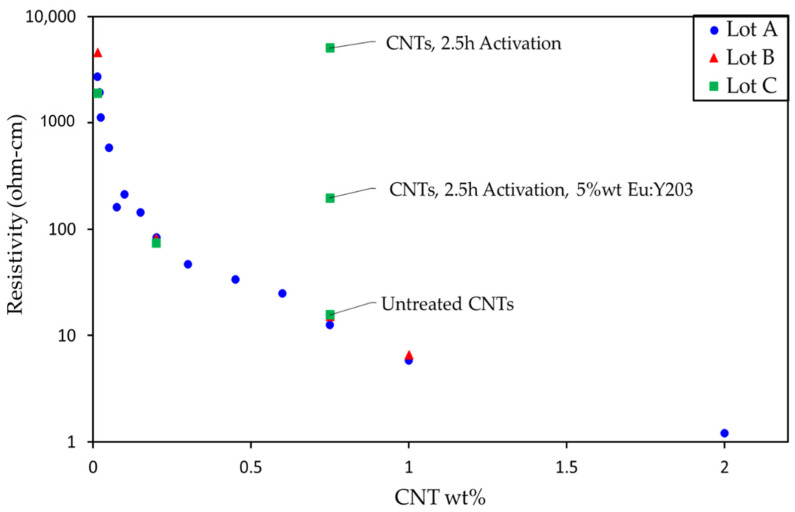
Measured electrical resistivity of current CNT pulp (Lot C) contrasted with past production runs (Lot A,B) with an applied current of 400 µA.

The electrical resistivity of the bulk nanocomposite must remain below 10–10^6^ and 10^6^–10^11^ Ohm-cm to maintain its applicability for EMI and ESD prevention applications, respectively [37]. Figure 9 presents the measured electrical resistivity of the epoxy composite as a function of %wt CNT loading. The samples generated in the present study span a large portion of the usable range for EMI and ESD applications. The composites based on 2.5 h activated CNTs had a resistivity roughly equal to past 0.014%wt CNT loadings of inactivated CNTs. However, once Eu:Y_2_O_3_ was included in the CNT epoxy, those values diminished, that is, the electrical conductivity increased.

**Figure 10:**
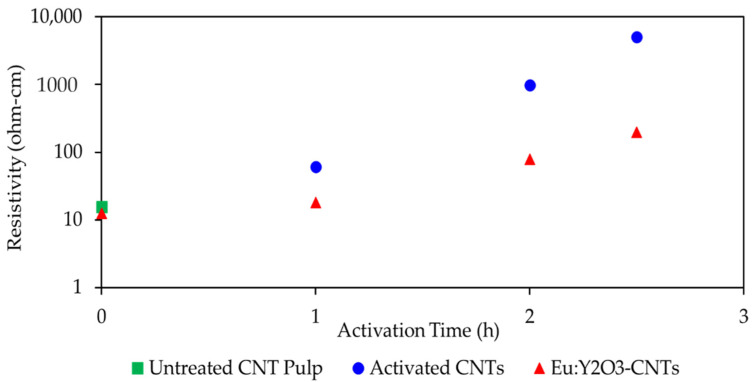
Effects of activation and inclusion of Eu:Y_2_O_3_ particles on electrical resistivity on a nanocomposite samples with 0.75%wt CNT loading.

Different activation times were explored, with each activated pulp subsequently subjected to synthesis of the Eu:Y_2_O_3_ nanoparticles. The resulting resistivity is plotted in Figure 10. The composite fabricated from untreated CNTs (0 h activation), 1, 2, and 2.5 h activation times resulted in resistivities of 15.67, 60.47, 982.7, and 5058 Ω-cm respectively. After synthesizing Eu:Y_2_O_3_, the untreated, 1 h, 2 h, and 2.5 h activated samples measured resistivities of 12.40, 18.14, 79.29, and 196.4 Ω-cm. Increases in resistivity during activation are attributed to the oxidation of the iron catalyst remaining from the CNT growth process into iron (III) oxide, a known electrical insulator [38]. Further, the activation process results in oxygen containing species in the form of ether C-O-C and quinone C=O groups along the CNT surface and likely plays a role in this decrease [39]. The resistivity improvements seen during the subsequent calcination are attributed to the reduction of the iron (III) oxide to small amounts of iron (II oxide) and iron. This is supported by the phase analysis presented in Figure 6.

**Figure 11:**
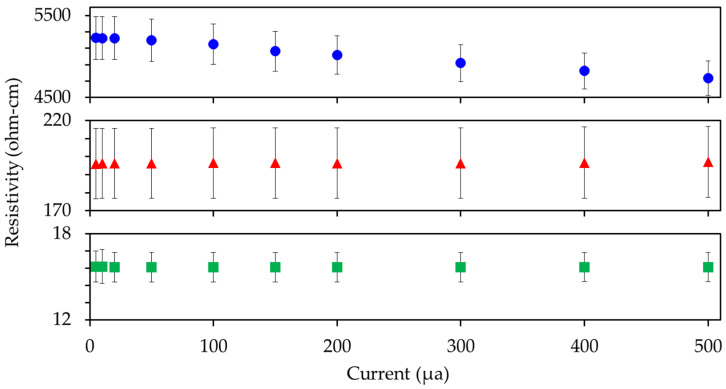
Electrical resistivity as a function of applied current for composites made with 0.75wt% CNTs at different phases of the preparing and Eu:Y_2_O_3_ synthesis process.

Figure 11 presents the resistivity as a function of applied current. Standard deviation across samples is stable at ~5–10% of measured resistivity. Decreasing resistivity under increased current application was seen for the activated CNT composite. A resistivity reduction of 9.4% across the full current range was recorded. The drop across the full scale in the untreated and Eu:Y_2_O_3_-CNT samples was <1%. Past research has documented similar behavior at the high resistivities associated with 0.014%wt CNT composites. This current dependence with increasing current application is ascribed to temperature increases due to resistive heating and irreversibility in the conductive path within the composite. Further discussion can be found in [1]. Notably, a reduction in resistivity (increased conductivity) is a positive attribute for the proposed applications.

### 3.3. Scanning Electron Microscopy and Micro-Computed Tomography

**Figure 12:**
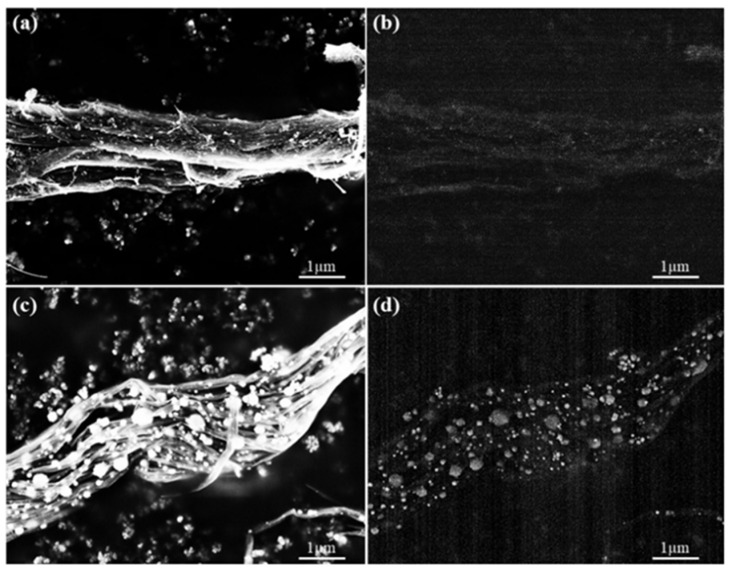
SEM imaging of CNT pulp: (**a**) SE image, (**b**) BSE image of untreated CNT pulp; (**c**) SE image, (**d**) BSE of Eu:Y_2_O_3_-CNT CNT pulp.

**Figure 13:**
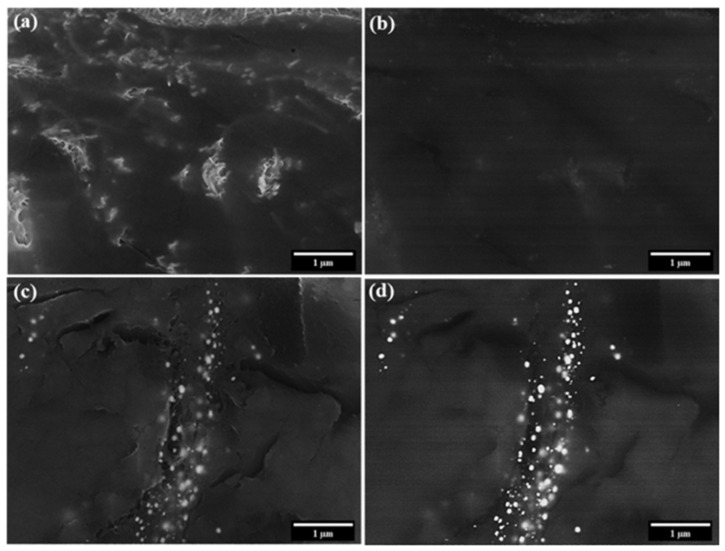
SEM imaging of 0.75%wt CNT nanocomposites made from: untreated CNTs (**a**) SE image (**b**) BSE image; Eu:Y_2_O_3_-CNTs (**c**) SE image (**d**) BSE image.

SEM imaging was conducted on untreated CNT pulp, Eu:Y_2_O_3_-CNT, and nanocomposites fabricated from those two samples. SE and BSE images of CNT pulp (no epoxy) and epoxy CNT nanocomposites are compared in Figure 12 and Figure 13.

For CNT pulp, the addition of Eu:Y_2_O_3_ results in easier identification of individual tubes due to the increased contrast of the colocated CNT, the sintered Fe particulates and the Eu:Y_2_O_3_ when conducting SE imaging (Figure 12a vs. Figure 12c). The larger particulates observed in Figure 12c correspond to the sintered Fe, while the Eu:Y_2_O_3_ is finely dispersed. BSE detection is improved with the addition of the Eu:Y_2_O_3_ oxide nanoparticles due to both the presence of heavier elements absent from the bare CNT pulp and that the Fe particulates remained at smaller sizes in the untreated CNT pulp. The contrast between Figure 12b,d is remarkable; Figure 12d shows a brighter tone for the Eu:Y_2_O_3_, previously identified by TEM as the finely dispersed particles, than for Fe. The CNT network is barely distinguishable in the background. BSE imaging of the untreated CNT bundles results in poor contrast.

Epoxy nanocomposites fabricated from untreated CNT pulp allow the visualization of a well-dispersed CNT network via SE electron imaging. BSE imaging results in poor contrast between the epoxy matrix and the CNT network in the same sample (Figure 13a,b). Eu:Y_2_O_3_-CNT nanocomposites show increased contrast in both imaging modes.

**Figure 14:**
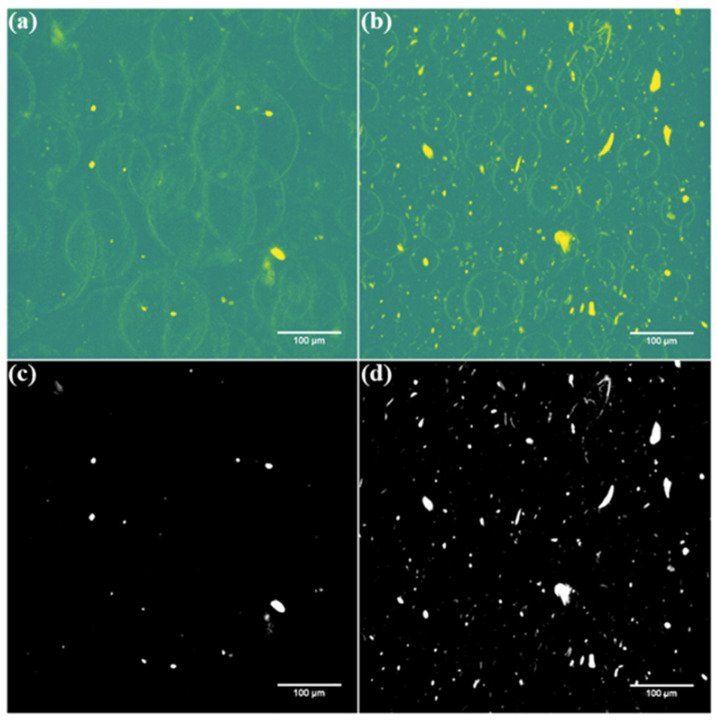
Micro-CT tomograms in high-contrast color revealing CNT network and pores and greyscale depicting only the CNT network: (**a**,**c**) untreated CNT nanocomposite; (**b**,**d**) Eu:Y_2_O_3_-CNT nanocomposite.

Micro-CT analysis was conducted on nanocomposites made from untreated CNTs and Eu:Y_2_O_3_ and generated 3D tomograms of internal porosity and CNT network. Videos featuring the 3D tomograms can be found online as Video S1 and Video S2. A plane-view of the captured volumes is presented in Figure 14. CNT loading was 0.75wt% in each case however, stark differences were seen between the composite with untreated CNT filler and the Eu:Y_2_O_3_-CNT composite (Figure 14a,b). CNT bundles were detected more readily in the Eu:Y_2_O_3_ sample and porosity differences were clear with average pore diameters of 113 nm and 50.2 nm for the untreated and Eu:Y_2_O_3_-CNTs, respectively. Image processing in Figure 14c,d allowed the pore outlines to drop out, making CNT bundle detection differences clearer. The contrast seen in the micro-CT scans corresponds to X-ray attenuation, which indicates the fraction of X-rays absorbed or scattered at each voxel. High atomic number elements such as Eu and Y attenuate X-rays more intensely compared to the epoxy matrix. Therefore, Eu:Y_2_O_3_-CNT manifest as high-contrast bright spots while pores in the matrix are shown as a circular outline with less intensity.

## 4. Conclusions

Eu:Y_2_O_3_ was synthesized as distinct particles attached to CNT walls. The addition of Eu:Y_2_O_3_ indirectly resulted in an increase in bulk resistivity when compared to the untreated state. However, nanocomposite resistivity remained well within the required limits for its intended EMI and ESD applications.

Photoluminescence of the Eu:Y_2_O_3_-CNTs under DUV excitation was documented with a resulting red-orange emission wavelength of 611 nm. The utility of luminescence in relation to the Eu:Y_2_O_3_-CNT nanocomposite was not tested. Future work could focus on developing imaging and nondestructive techniques exploiting the photoluminescence within a cured composite.

Through this presence of heavier rare-earth elements, i.e., yttrium and europium, significant contrast improvements were realized in the SEM BSE imaging, potentially allowing the detection of tube bundles more readily and at greater depths. Similarly, the micro-CT scans benefited from Eu:Y_2_O_3_ inclusion and improvements to three-dimensional visualization of the CNT network through the reconstructed images resulted.

## Supplementary Materials

The following are available online at https://www.mdpi.com/article/10.3390/nano11092168/s1, Video S1: 3D tomogram of untreated CNT composite, Video S2: 3D tomogram of Eu:Y_2_O_3_-CNT composite.

## References

[1] Earp B., Phillips J., Grbovic D., Vidmar S., Porter M., Luhrs C.C. (2020). Impact of Current and Temperature on Extremely Low Loading Epoxy-CNT Conductive Composites. Polymers.

[2] Bellucci S., Balasubramanian C., Micciulla F., Rinaldi G. (2007). CNT Composites for Aerospace Applications. J. Exp. Nanosci..

[3] Du J.-H., Bai J., Cheng H.-M. (2007). The Present Status and Key Problems of Carbon Nanotube Based Polymer Composites. Express Polym. Lett..

[4] Harris P.J.F. (2004). Carbon Nanotube Composites. Int. Mater. Rev..

[5] Kausar A., Rafique I., Muhammad B. (2016). Review of Applications of Polymer/Carbon Nanotubes and Epoxy/CNT Composites. Polym.-Plast. Technol. Eng..

[6] Kumar P., Maiti U.N., Sikdar A., Das T.K., Kumar A., Sudarsan V. (2019). Recent Advances in Polymer and Polymer Composites for Electromagnetic Interference Shielding: Review and Future Prospects. Polym. Rev..

[7] Park S.-H., Ha J.-H. (2019). Improved Electromagnetic Interference Shielding Properties Through the Use of Segregate Carbon Nanotube Networks. Materials.

[8] Spitalsky Z., Tasis D., Papagelis K., Galiotis C. (2010). Carbon Nanotube–Polymer Composites: Chemistry, Processing, Mechanical and Electrical Properties. Prog. Polym. Sci..

[9] De Volder M.F.L., Tawfick S.H., Baughman R.H., Hart A.J. (2013). Carbon Nanotubes: Present and Future Commercial Applications. Science.

[10] Yang Y., Gupta M.C., Dudley K.L. (2007). Studies on Electromagnetic Interference Shielding Characteristics of Metal Nanoparticle- and Carbon Nanostructure-Filled Polymer Composites in the Ku-Band Frequency. Micro Nano Lett..

[11] Khan W., Sharma R., Saini P., Berber M., Hafez I.H. (2016). Carbon Nanotube-Based Polymer Composites: Synthesis, Properties and Applications. Carbon Nanotubes—Current Progress of Their Polymer Composites.

[12] Pantano A., Montinaro N., Cerniglia D., Micciulla F., Bistarelli S., Cataldo A., Bellucci S. (2019). Novel Non-Destructive Evaluation Technique for the Detection of Poor Dispersion of Carbon Nanotubes in Nanocomposites. Compos. Part B Eng..

[13] Li W., Buschhorn S.T., Schulte K., Bauhofer W. (2011). The Imaging Mechanism, Imaging Depth, and Parameters Influencing the Visibility of Carbon Nanotubes in a Polymer Matrix Using an SEM. Carbon.

[14] Finnie P., Kaminska K., Homma Y., Austing D.G., Lefebvre J. (2008). Charge Contrast Imaging of Suspended Nanotubes by Scanning Electron Microscopy. Nanotechnology.

[15] Loos J., Alexeev A., Grossiord N., Koning C.E., Regev O. (2005). Visualization of Single-Wall Carbon Nanotube (SWNT) Networks in Conductive Polystyrene Nanocomposites by Charge Contrast Imaging. Ultramicroscopy.

[16] Lillehei P.T., Kim J.-W., Gibbons L.J., Park C. (2009). A Quantitative Assessment of Carbon Nanotube Dispersion in Polymer Matrices. Nanotechnology.

[17] Kovacs J.Z., Andresen K., Pauls J.R., Garcia C.P., Schossig M., Schulte K., Bauhofer W. (2007). Analyzing the Quality of Carbon Nanotube Dispersions in Polymers Using Scanning Electron Microscopy. Carbon.

[18] Earp B., Simpson J., Phillips J., Grbovic D., Vidmar S., McCarthy J., Luhrs C. (2019). Electrically Conductive CNT Composites at Loadings below Theoretical Percolation Values. Nanomaterials.

[19] Chang N.C. (1963). Fluorescence and Stimulated Emission from Trivalent Europium in Yttrium Oxide. J. Appl. Phys..

[20] Chang N.C., Gruber J.B. (1964). Spectra and Energy Levels of Eu^3+^ in Y_2_O_3_. J. Chem. Phys..

[21] Blasse G., Grabmaier B.C. (1994). Luminescent Materials.

[22] Mariscal-Becerra L., Vázquez-Arreguín R., Balderas U., Carmona-Téllez S., Murrieta Sánchez H., Falcony C. (2017). Luminescent Characteristics of Layered Yttrium Oxide Nano-Phosphors Doped with Europium. J. Appl. Phys..

[23] Wakefield G., Holland E., Dobson P.J., Hutchison J.L. (2001). Luminescence Properties of Nanocrystalline Y2O3:Eu. Adv. Mater..

[24] Schmechel R., Kennedy M., von Seggern H., Winkler H., Kolbe M., Fischer R.A., Xaomao L., Benker A., Winterer M., Hahn H. (2001). Luminescence Properties of Nanocrystalline Y_2_O_3_:Eu^3+^ in Different Host Materials. J. Appl. Phys..

[25] Das G.K. (2010). Rare Earth Doped Nanomaterials as Potential Contrast Agents for Optical/Magnetic Resonance Imaging.

[26] Yang H., Zhang D., Shi L., Fang J. (2008). Synthesis and Strong Red Photoluminescence of Europium Oxide Nanotubes and Nanowires Using Carbon Nanotubes as Templates. Acta Mater..

[27] Technologies N. Nanocomp Technologies’ Products|Dispersed Products. https://www.miralon.com/dispersed-products.

[28] LOCTITE 9396aero. https://www.henkel-adhesives.com/vn/en/product/adhesives/loctite_ea_9396_aero.html.

[29] Tomviz for Tomographic Visualization of Nanoscale Materials. https://tomviz.org/.

[30] Melnikov P., Nascimento V.A., Consolo L.Z.Z., Silva A.F. (2013). Mechanism of Thermal Decomposition of Yttrium Nitrate Hexahydrate, Y(NO_3_)_3_·6H_2_O and Modeling of Intermediate Oxynitrates. J. Therm. Anal. Calorim..

[31] Melnikov P., Arkhangelsky I.V., Nascimento V.A., de Oliveira L.C.S., Silva A.F., Zanoni L.Z. (2017). Thermal Properties of Europium Nitrate Hexahydrate Eu(NO_3_)_3_·6H_2_O. J. Therm. Anal. Calorim..

[32] Jack K.H., Goodeve C.F. (1951). The Iron-Nitrogen System: The Preparation and the Crystal Structures of Nitrogen-Austenite(γ) and Nitrogen-Martensite(α’). Proc. R. Soc. Lond. Ser. Math. Phys. Sci..

[33] Tessier F., Navrotsky A., Niewa R., Leineweber A., Jacobs H., Kikkawa S., Takahashi M., Kanamaru F., DiSalvo F.J. (2000). Energetics of Binary Iron Nitrides. Solid State Sci..

[34] Hirai H., Kondo K. (1991). Modified Phases of Diamond Formed Under Shock Compression and Rapid Quenching. Science.

[35] Wen B., Zhao J.J., Li T.J. (2007). Synthesis and Crystal Structure of N-Diamond. Int. Mater. Rev..

[36] Wen B., Zhao J., Li T., Dong C., Jin J. (2005). N-Diamond from Catalysed Carbon Nanotubes: Synthesis and Crystal Structure. J. Phys. Condens. Matter.

[37] Pang H., Xu L., Yan D.-X., Li Z.-M. (2014). Conductive Polymer Composites with Segregated Structures. Prog. Polym. Sci..

[38] Mochizuki S. (1977). Electrical Conductivity of α-Fe_2_O_3_. Phys. Status Solidi A.

[39] Chen X., Tang X.-Z., Liang Y.N., Cheah J.W., Hu P., Hu X. (2016). Controlled Thermal Functionalization for Dispersion Enhancement of Multi-Wall Carbon Nanotube in Organic Solvents. J. Mater. Sci..

